# A Non-Human Primate Model for Gluten Sensitivity

**DOI:** 10.1371/journal.pone.0001614

**Published:** 2008-02-20

**Authors:** Michael T. Bethune, Juan T. Borda, Erin Ribka, Michael-Xun Liu, Kathrine Phillippi-Falkenstein, Ronald J. Jandacek, Gaby G. M. Doxiadis, Gary M. Gray, Chaitan Khosla, Karol Sestak

**Affiliations:** 1 Department of Biochemistry, Stanford University, Stanford, California, United States of America; 2 Tulane National Primate Research Center, Covington, Louisiana, United States of America; 3 Department of Pathology and Laboratory Medicine, University of Cincinnati, Cincinnati, Ohio, United States of America; 4 Biomedical Primate Research Centre, Rijswijk, The Netherlands; 5 Department of Medicine, Stanford University, Stanford, California, United States of America; 6 Department of Chemistry, Stanford University, Stanford, California, United States of America; 7 Department of Chemical Engineering, Stanford University, Stanford, California, United States of America; 8 Department of Microbiology and Immunology, Tulane University School of Medicine, New Orleans, Louisiana, United States of America; Instituto Oswaldo Cruz and FIOCRUZ, Brazil

## Abstract

**Background and Aims:**

Gluten sensitivity is widespread among humans. For example, in celiac disease patients, an inflammatory response to dietary gluten leads to enteropathy, malabsorption, circulating antibodies against gluten and transglutaminase 2, and clinical symptoms such as diarrhea. There is a growing need in fundamental and translational research for animal models that exhibit aspects of human gluten sensitivity.

**Methods:**

Using ELISA-based antibody assays, we screened a population of captive rhesus macaques with chronic diarrhea of non-infectious origin to estimate the incidence of gluten sensitivity. A selected animal with elevated anti-gliadin antibodies and a matched control were extensively studied through alternating periods of gluten-free diet and gluten challenge. Blinded clinical and histological evaluations were conducted to seek evidence for gluten sensitivity.

**Results:**

When fed with a gluten-containing diet, gluten-sensitive macaques showed signs and symptoms of celiac disease including chronic diarrhea, malabsorptive steatorrhea, intestinal lesions and anti-gliadin antibodies. A gluten-free diet reversed these clinical, histological and serological features, while reintroduction of dietary gluten caused rapid relapse.

**Conclusions:**

Gluten-sensitive rhesus macaques may be an attractive resource for investigating both the pathogenesis and the treatment of celiac disease.

## Introduction

Celiac disease is an inheritable enteropathy caused by dietary gluten from wheat, barley, and rye [1]. Its clinical manifestations are variable, but commonly include persistent diarrhea, abdominal discomfort, bloating, and fatigue. In some celiac patients, a pruritic, vesicular skin rash called dermatitis herpetiformis accompanies gastrointestinal damage [2], [3]. The deleterious immune response underlying these symptoms is mediated by intestinal lymphoid tissue in response to proteolytically resistant gluten peptides bound to human leukocyte antigen (HLA) DQ2, a class II major histocompatibility complex (MHC) molecule associated with over 90% of diagnosed celiac patients [4], [5]. Gluten elicits both a T cell and a B cell response in patients with untreated celiac disease [6]. A biopsy of the intestinal lesion showing characteristic villus blunting, crypt hyperplasia, and intraepithelial lymphocytosis remains the gold standard for diagnosis [7], though circulating antibodies against gliadin (the alcohol-soluble fraction of gluten) [8], endomysium and/or endogenous transglutaminase 2 (TG2) [9] are now widely used as specific indicators of disease. Such serological tests have established the prevalence of celiac disease to be as high as 1∶100 in certain populations, although the condition remains under-diagnosed [10], [11]. Untreated celiac disease is associated with increased morbidity and mortality, while strict dietary exclusion of gluten constitutes an effective treatment [12].

Clinical, immunological, genetic, and biochemical studies have greatly expanded our understanding of the progression of celiac disease [6], but the elucidation of several critical inquiries remaining in celiac disease research would be greatly facilitated by a suitable animal model of gluten sensitivity. For instance, it is not known how gluten peptides are transported intact across the mucosal epithelium for presentation to the underlying lymphoid tissue, or how disease state affects this phenomenon. In fact, the detection of transepithelial transport of a chemically-defined gluten peptide *in vivo* has not been reported, though it is presumed such an event is prerequisite to disease. The study of whether there is a primary defect in gut permeability in human celiac patients is hampered by the difficulty of ensuring adherence to a gluten-free diet in the midst of ubiquitous gluten-containing human foodstuffs. However, such studies could be conducted in an animal model in which dietary consumption of gluten could be strictly controlled. The challenge celiac patients face in maintaining a gluten-free diet also significantly impacts their quality of life, necessitating the development of alternative (or adjunct) non-dietary therapies [13]. A particularly promising route is the use of oral glutenases [14], [15], proteases capable of detoxifying ingested gluten, but here again an animal model of gluten sensitivity is needed to make a preclinical determination on the efficacy of such therapeutic interventions. Thus, animal models of gluten sensitivity would enable the study of both fundamental and practical questions related to celiac disease.

Here, we identify juvenile rhesus macaques (*Macaca mulatta*) as a model exhibiting clinical, serological, and histological signs and symptoms of gluten sensitivity. In an accompanying report, we describe additional studies demonstrating the potential of this model for studying gluten peptide transport and therapeutic intervention in celiac disease [16].

## Methods

### Rhesus macaques

Retrovirus-free (simian immunodeficiency virus, simian retrovirus, and simian T-cell leukemia virus) rhesus macaques (*Macaca mulatta*) of all ages, and both sexes were used in initial epidemiological pre-screening of diarrhea as the cause of morbidity in Tulane National Primate Research Center (TNPRC) breeding colony macaques (n = 2,820). A subset of these animals (n = 83) with clinical symptoms of non-infectious, chronic diarrhea of idiopathic origin was identified and tested for the presence of anti-gliadin antibodies (AGA). An AGA+ juvenile macaque (FH09) with chronic diarrhea, dehydration, and stomach distention was selected for treatment with a gluten-free diet. In addition, a clinically healthy, age-matched juvenile macaque (FR26) underwent identical dietary treatment. Animals undergoing dietary treatment were housed for 10 months under biosafety level two conditions in accordance with the standards of the Association for Assessment and Accreditation of Laboratory Animal Care. Investigators adhered to the Guide for the Care and Use of Laboratory Animals prepared by the National Research Council.

### Gluten-containing and gluten-free diets

Four different types of diets were formulated and used in this study in collaboration with Purina, Inc. Diet #1 corresponded to a commercially available monkey chow (5K63) that is routinely used to feed captive rhesus macaques and that fulfills all nutritional requirements, as specified by its manufacturer (PMI Nutrition Int., LLC.). This diet contained 20% (by weight) of crude protein including oats and gluten sources such as ground wheat, 5% of fat, and 10% of crude fiber. Diet #2 (reduced gluten diet) contained all the nutrients at levels identical with diet #1, except proteins from wheat gluten sources were omitted. Diet #3 (gluten-free diet, 5A7Q) contained all nutrients at levels identical with diet #1, except proteins from all gluten sources were omitted. Diet #4 (high fat gluten-free diet with inclusion of 1.055% (w/w) sucrose polybehenate (P&G, 5B3W)) contained all nutrients at levels identical with diet #3, except it contained 21.1% fat and the non-absorbable, lipophilic marker, sucrose polybehenate, for evaluation of steatorrhea as described in detail elsewhere [17]. Animals would typically consume 4% of their body weight daily (e.g. 160 g of food for a 4 kg animal).

### Clinical evaluation of gluten sensitivity

Clinical data were recorded daily for the duration of the 8–10 month dietary treatment periods. Average weekly values were calculated as means±standard deviation of 7-day intervals. Stool samples obtained from each macaque at the time of study assignment were tested according to our protocols [18] to confirm that no infectious pathogens were contributing to clinical symptoms of illness. Criteria that were used in blinded clinical scoring (1–6 scale) of gluten sensitivity were scaled relative to “clinically normal”, age-matched controls, i.e. score of 1. Score 2 corresponded to beginning of diarrhea, i.e. pasty stools. Score 3 corresponded to semi-liquid stools and decreased activity. Score 4 corresponded to liquid stools, decreased activity, moderate dehydration and “balloon” stomach. Score 5 corresponded to liquid stools, depression, severe dehydration and balloon stomach. Score 6 would correspond to a moribund animal where prompt euthanasia is recommended. Such a severe score was never reached.

### Veterinary procedures: peripheral blood and small intestinal biopsy sample collections

Peripheral blood samples (1 ml) were collected from the femoral vein, and plasma was harvested. Collected samples were stored at −80°C until analyzed for anti-gliadin and anti-TG2 antibodies. Blood samples were obtained from A) FH09 and FR26 macaques in bi-weekly intervals during the entire study period, and B) from colony macaques during annual inventories.

Endoscope-guided pinch biopsies were collected by a TNPRC veterinary surgeon from the distal duodenum of gluten-sensitive FH09 and of control FR26 at 0, 27 and 37 weeks after the first diet switch (diet # 1 to diet # 2). Ten pieces (each ∼3 mm^3^) were obtained from each animal and fixed in formalin for routine histology and colocalization of immunological markers. In addition, 5 grams of stool samples were collected from FH09 and FR26 at weeks 27 and 37 after feeding them diet #4 for 4 consecutive days. As approved by our IACUC protocol and confirmed by past experiments, macaques tolerate intestinal pinch biopsies with no adverse effects. Plasma and biopsy collections were performed with sedated and anesthetized animals.

Archived post-mortem biopsies from 40 necropsy macaques were collected and fixed immediately following death. No autolytic tissues were included.

### Indirect ELISA for AGA and anti-TG2 antibodies

Relative plasma levels of anti-gliadin and anti-TG2 antibodies were determined by indirect ELISA. For anti-gliadin antibody tests, 20 mg/ml gliadin (Sigma) was digested in 0.01 M HCl for 60 min at 37°C with 0.6 mg/ml pepsin (American Laboratories). The reaction was then adjusted to pH 6.0 with Na_2_HPO_4_ and 0.375 mg/ml trypsin (Sigma) was added to further digest the gliadin for 120 min at 37°C. The reaction was quenched by boiling 10 min and frozen at −20°C until use. Pepsin-trypsin digested gliadin was diluted to 20 µg/ml in coating solution (50 mM sodium carbonate/bicarbonate buffer, pH 9.6, 0.02% NaN_3_) and 200 µl/well was incubated overnight at 4°C in 96-well microtiter plates (Nunc Maxisorp). For anti-TG2 antibody tests, 2–20 µg/ml recombinant human TG2, expressed and purified as previously described [19], was used to coat the plates instead of pepsin-trypsin digested gliadin. Antigen-coated plates were washed three times with 1×PBS, pH 7.4, 0.05% Tween-20 prior to blocking and between all subsequent steps. Plates were blocked with 200 µl blocking buffer (1xPBS, pH 7.4, 0.05% Tween-20, 3% BSA) for 1 h at room temperature. Plasma samples were diluted 1∶1000 in blocking buffer and 200 µl/well was incubated overnight at 4°C. Secondary antibody-alkaline phosphatase conjugates (rabbit anti-monkey IgG (Sigma) or goat anti-monkey IgA (RDI Fitzgerald)) were diluted 1∶250 in blocking buffer and 200 µl/well was incubated 3 h at room temperature. Freshly prepared substrate solution (5 mg/ml pNPP, 50 mM sodium carbonate/bicarbonate buffer, pH 9.8, 1 mM MgCl_2_, 0.02% NaN_3_) was added (200 µl/well) and the absorbance at 405 nm was measured every 6 seconds for 5 minutes. The initial rate (mA_405_/min) in each well was determined from 20 data points. All samples were analyzed in triplicate. Thresholds for identifying samples as positive for anti-gliadin IgG (20.5 mA_405_/min) and IgA (2.10 mA_405_/min) were determined by optimizing for sensitivity and specificity through receiver-operator characteristic (ROC) analysis. The areas under the anti-gliadin IgG and IgA ROC curves were 0.627 and 0.874, respectively.

### Histopathological examination and morphometric evaluation of villus blunting

Distal duodenum tissue samples collected from gluten-sensitive FH09 and control FR26 macaques were processed as described in detail previously [18]. Briefly, five 3 mm^3^ tissue pieces were fixed in 10% neutral buffered formalin, sectioned at 6 µm, stained with hematoxylin and eosin (H&E), and viewed under regular light microscopy at 100–200×. The villus height/crypt depth (V∶C) ratios were obtained from linear measurements of 5 villus heights, divided by the corresponding crypt depths. The linear measurement of the villus height was made from the top of the villi to the mouth of the crypt of Lieberkuhn. The crypt depth was measured as the distance from the mouth of the crypt of Lieberkuhn to the upper border of intestinal lamina muscularis. Typical lesions that we expected to find in cases of enteropathy included duodenal villus blunting, reduced V:C ratio, crypt hyperplasia and dilatation, and increased number of IELs. None of these changes were expected in a normal, control animal. Archived samples from necropsy animals were evaluated similarly. For this purpose, we analyzed historical samples of hematoxylin/eosin-stained distal duodenum and proximal jejunum tissues from 40 TNPRC rhesus macaques with chronic diarrhea. Evaluations were performed blinded to the sample source.

### Visualization of IELs in situ

The technique that was used with some modifications is described in detail elsewhere [20], [21]. Briefly, five pieces of pinch (3 mm^3^) biopsies were sectioned at 6 µm thick and subjected to immunohistochemistry. Sections were incubated with the primary, cell-type specific antibody for 60 minutes (CD3, polyclonal, or CD103, monoclonal, clone Ber-ACT8, both Dako) at room temperature followed by biotinylated goat anti-rabbit or horse anti-mouse (Vector Laboratories) secondary antibodies, respectively. Finally, sections were incubated with avidin-biotin-complex (ABC, Elite, Vector Laboratories), and the reaction was visualized with 3,3′-diaminobenzidine as the chromogen (Dako). As a negative control, serial sections were processed identically using equivalent concentrations of irrelevant primary antibodies of the same isotype. Visualization was performed using a Leica DMLB microscope with a SPOT insight digital camera (Digital Instruments) interfaced to Image-Pro Plus image analysis software (Media Cybernetics). The CD3+ T cells in epithelium (and subepithelium) were visualized in brown color. This staining allows automated color enhancement and identification of positive cells in specified areas of interest.

### Measurement of malabsorptive steatorrhea

At week 27, following treatment with a gluten-free diet for ∼11 weeks, gluten-sensitive FH09 was placed for 4 days on diet #4, which enabled measurement of fat content in 5 g stool samples collected at 32, 48, 56 and 72 hours post diet #4 initiation. Fatty acid absorption, expressed as the percent of ingested dietary fat, was calculated as described elsewhere [17]. Tabulated values are shown for 56 and 72 hours, since it took >48 hours post diet #4 initiation for fat absorption levels to stabilize. Measurement of fat content in stools was repeated in gluten-sensitive FH09 and control FR26 macaques at week 37 when they were challenged with gluten-containing diet #1.

### Statistical evaluation

Statistical differences between the level of AGA and between V:C ratios in gluten-sensitive versus control macaques were determined by the Kruskal-Wallis Test for non-parametric comparisons using at least three independent measurements from each animal. A p value of <0.05 was considered significant.

## Results

### Prevalence of idiopathic diarrhea

To determine the prevalence of chronic diarrhea as a cause of morbidity in captive rhesus macaques, we conducted a retrospective epidemiological survey of 2,820 breeding colony animals housed at the Tulane National Primate Research Center (TNPRC). These animals were retrovirus-free (simian immunodeficiency virus, simian retrovirus, and simian T cell leukemia virus), of all ages, and of both sexes. The overall prevalence of diarrhea in the population was 7.5/100 animals, with highest rates observed among yearlings (16.1/100) and 2 to 4 year olds (10.5/100) (Table 1). In these juvenile animals, diarrhea was in some instances accompanied by bloating (balloon stomach) and skin rash/blistering, clinical presentations that are characteristic of classic human celiac disease and of the dermatologic manifestation of celiac disease, dermatitis herpetiformis [2], [3].

**Table 1:** Morbidity for rhesus macaques (*Macaca mulatta*) at TNPRC

Age Category	Population	Presenting Cause		Diarrhea Rate per 100 animals
		Diarrhea	Other	
Infant	491	22	47	4.5
Yearling	471	76	23	16.1
2 to 4	737	77	99	10.5
4 to 6	505	17	109	3.4
6 to 8	279	12	88	4.3
8 to 10	88	1	25	1.1
10 to 12	75	0	23	0
12 to 14	57	3	6	5.3
14 to 16	55	3	10	5.5
16 to 18	19	1	5	5.3
18 to 20	13	0	1	0
20 & Up	32	0	6	0
Total	2820	212	442	7.5

### Histology

**Figure 1:**
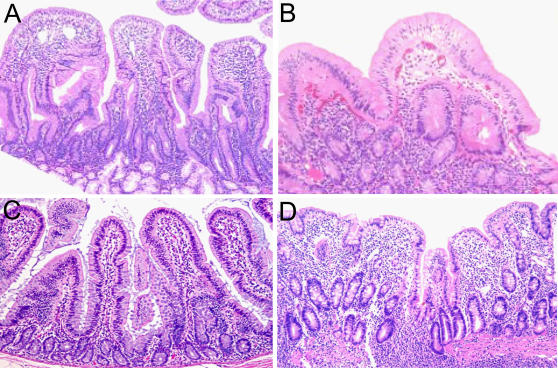
Histopathology of the small intestine. H&E-stained tissue sections of duodenum and proximal jejunum from rhesus macaques with idiopathic diarrhea. (A) Normal control duodenum from an age-matched rhesus macaque illustrating characteristic morphology of the villi. 100× magnification. (B) Enteropathy of duodenum. Diffuse enteritis characterized by shortening of villi, severe lymphocytic and plasmacytic infiltration of the lamina propria, and vacuolar degeneration of the epithelium. 100× magnification. (C) Normal control jejunum from an age-matched rhesus macaque. 100× magnification. (D) Enteropathy of jejunum. The mucosa appears flat with marked blunting of villi and dense infiltration of lamina propria by mononuclear cells. 100× magnification.

Based on these clinical symptoms, we hypothesized that macaques suffering from chronic diarrhea may also exhibit histological lesions that in human patients are indicative of celiac disease. We therefore evaluated archived tissues from 40 available necropsy macaques with histories of chronic enteropathy and clinical diarrhea of non-infectious, idiopathic origin, searching specifically for the presence of partial or complete villus blunting, crypt hyperplasia and inflammation. Villus flattening, crypt hyperplasia and intraepithelial lymphocytosis are typically present in human patients in advanced stages of celiac disease, while patients in earlier stages of the disease exhibit only lymphocytosis, with normal villus architecture or only partial villus atrophy [3], [22]. Villus blunting, crypt hyperplasia, and intraepithelial lymphocytosis were identified in 12.5% (5/40) of these animals (Figure 1). Morphometric evaluation of duodenal villus height:crypt depth (V:C) ratios confirmed that V:C ratios were significantly lower in lesioned animals (0.6±0.1) relative to controls (1.7±0.2)(p<0.01, Mann-Whitney Test). This 2.8-fold average decrease in V:C ratio is similar in magnitude to that defined as partial villus atrophy in celiac disease [23]. The histology in these macaques suffering from chronic diarrhea of non-infectious origin therefore resembles that observed during advanced stages of celiac disease.

### Serology

A critical environmental trigger in celiac disease is dietary gluten from wheat as well as related protein from barley and rye. The primary pathogenic components of wheat gluten are gliadin proteins, and serological tests for the presence of elevated IgG and IgA anti-gliadin antibodies (AGA) are respectively 89% and 82% sensitive and 66% and 90% specific for celiac disease in human patients [24]. As captive rhesus macaques are fed a diet containing gluten, a protein source that is not a staple in a wild macaque's diet, we conducted a serological survey to determine if dietary gluten might contribute to the high prevalence of gastrointestinal illness in these primates. A selection of 83 retrovirus-free rhesus macaques exhibiting chronic diarrhea of non-infectious etiology were tested for IgG and IgA AGA by indirect ELISA (Table 2). Both juvenile (≤4 years; n = 66) and adult (>4 years; n = 17) subgroups exhibited elevated IgG and IgA AGA levels relative to healthy controls (≤4 years; n = 11), indicating that gluten in commercial monkey chow (diet #1) elicits a humoral immune response in some rhesus macaques. The proportion of symptomatic animals with elevated IgG was twice as high in juveniles (46/66) than in adults (6/17), whereas nearly all symptomatic animals exhibited elevated IgA against gliadin (63/66 juveniles and 17/17 adults). In healthy juveniles, the corresponding proportions were 3/11 IgG+ and 1/11 IgA+.

**Table 2:** Anti-gliadin antibodies (AGA) in TNPRC rhesus macaques with histories of clinical diarrhea

		History of diarrhea	IgG AGA		IgA AGA	
Age Category	Total		+	−	+	−
Healthy juveniles (≤4 years)	11	No	3	8	1	10
Symptomatic juveniles (≤4 years)	66	Yes	46	20	63	3
Symptomatic adults (>4 years)	17	Yes	6	11	17	0
Total (Symptomatic)	83		52	31	80	3

A subset of 15 AGA+ animals (including those with the highest AGA levels) and all healthy controls were further tested for the presence of anti-TG2 antibodies, which are known to be more specific (97%) and sensitive (94%) indicators of celiac disease than AGA [24]. Although three of these AGA+ individuals exhibited elevated anti-TG2 antibodies relative to the controls, the increase was small (∼2-fold), and did not correlate with AGA levels (data not shown).

### Removal of dietary gluten improves clinical diarrhea, histology, and serology

The presence of AGA in enteropathic rhesus macaques does not necessarily identify dietary gluten as a causative agent in the disease. Since gluten is highly resistant to gastrointestinal proteolysis [25], damage to the macaque gut from other factors (e.g. bacterial or viral infection) could increase the mucosal permeability toward luminal gluten, leading to a humoral immune response against gluten absent any direct pathogenic effect. In celiac disease, a definitive diagnosis requires that histological lesions are present when the patient is on a gluten-containing diet, and that unequivocal clinical and histological improvement occurs upon adherence to a gluten-free diet [2], [7]. To determine if dietary gluten is a trigger of the clinical and serological condition described above in rhesus macaques, we successively administered 2 modified diets (reduced gluten and gluten-free) to FH09, an AGA+ juvenile macaque with chronic diarrhea, as well as to FR26, an age-matched AGA- control macaque that experienced no gluten sensitivity. Clinical, histological, and serological markers of enteropathy were monitored over time to assess recovery in FH09.

**Figure 2:**
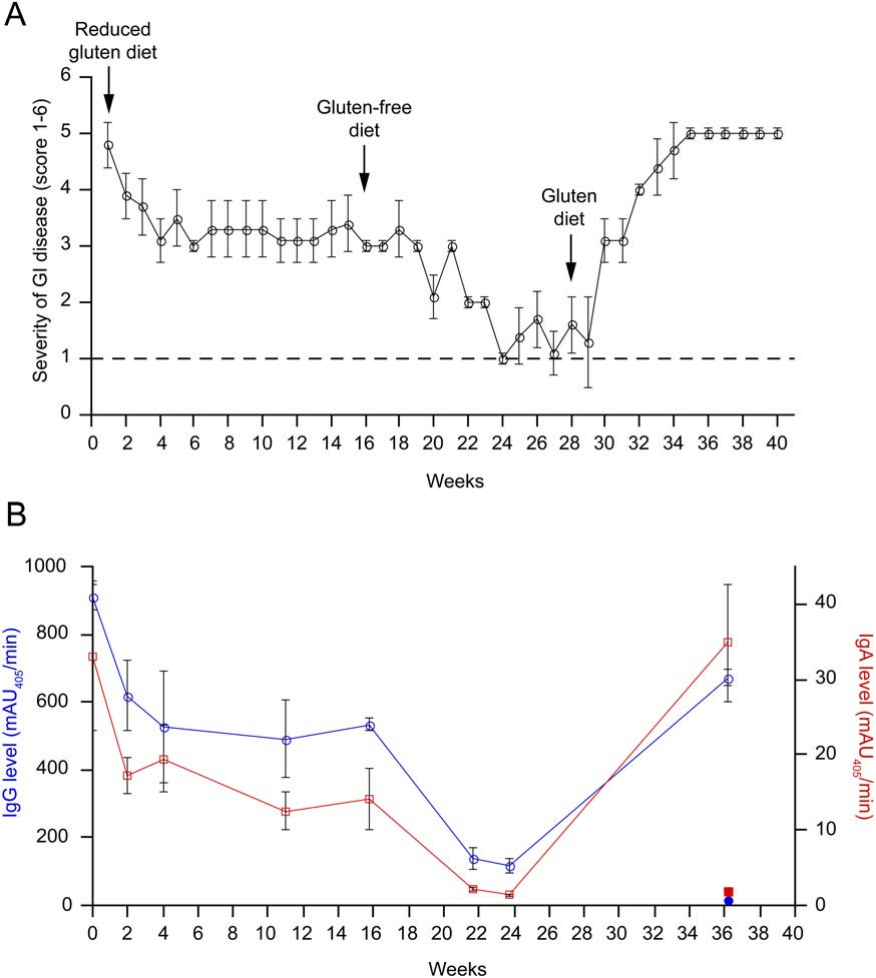
Gluten dependence of clinical symptoms and serology in gluten-sensitive rhesus macaque FH09. (A) Gastrointestinal symptoms in gluten-sensitive FH09 improve with sequential administration of reduced gluten and gluten-free diets, but return upon reintroduction of dietary gluten (diet changes indicated by vertical arrows). Criteria that were used in clinical scoring of gluten sensitivity in the juvenile macaque FH09 were scaled relative to the healthy, age-matched control, FR26 (score 1, indicated by dotted line). Score 2 corresponded to beginning of diarrhea, e.g. pasty stools. Score 3 corresponded to semi-liquid stools and decreased activity. Score 4 corresponded to liquid stools, decreased activity, moderate dehydration and “balloon” stomach. Score 5 corresponded to liquid stools, depression, severe dehydration and balloon stomach. Score 6 would correspond to a moribund animal where prompt euthanasia is recommended. Each datapoint represents the mean of 7 daily measurements taken over the course of the indicated week. Standard deviations are indicated by error bars. (B) Anti-gliadin IgG (blue; open circles) and IgA (red; open squares) return to baseline with dietary exclusion of gluten, but are elevated following reintroduction of dietary gluten. The level of anti-gliadin IgG (blue; closed circle) and IgA (red; closed square) in control FR26 are shown for comparison. Each datapoint represents the mean of triplicate measurements. Standard deviations are indicated by error bars.

On a gluten-containing diet, FH09 presented with chronic diarrhea, dehydration, distended stomach, and physical inactivity, as well as elevated plasma levels of both IgG and IgA against gliadin (Figure 2). Within 6 days of changing to a reduced gluten diet (diet #2), clinical symptoms of diarrhea in FH09 improved (Figure 2A), and by 4 weeks its AGA levels had reduced by half (Figure 2B). However, over the subsequent 12 weeks on this diet, FH09 still exhibited occasional diarrhea, distended stomach, and mild dehydration, and thus did not improve to an asymptomatic stage (clinical score of 1). Additionally, levels of AGA in FH09 remained significantly elevated above those observed in FR26. Therefore, after 16 weeks on a reduced gluten diet, FH09 and FR26 were reassigned to an entirely gluten-free diet (diet #3). Within 8 weeks of this change, FH09 clinically recuperated, no longer exhibiting symptoms of disease such as diarrhea, dehydration, or abdominal distention (Figure 2A), and its IgG and IgA AGA further dropped to levels that were statistically indistinguishable from the healthy, age-matched juvenile macaque FR26 (Figure 2B). Moreover, dietary fat absorption in FH09 on a gluten-free diet was ≥80%, which was comparable (p<0.05) to the level of dietary fat absorption measured in FR26 (Table 3).

**Table 3:** Fat absorption in FH09 (gluten-sensitive) and FR26 (control) macaques

		Behenate marker absorption (%)	
Animal	Experiment	56 hours on diet #4	72 hours on diet #4
FH09	week 27 (gluten-free)	80.2 ± 6.9	78.4 ± 4.9
FH09	week 37 (gluten challenge)	67.0 ± 1.4	67.8 ± 1.1
FR26	week 37 (gluten challenge)	92.2 ± 1.1	89.1 ± 0.1

**Figure 3:**
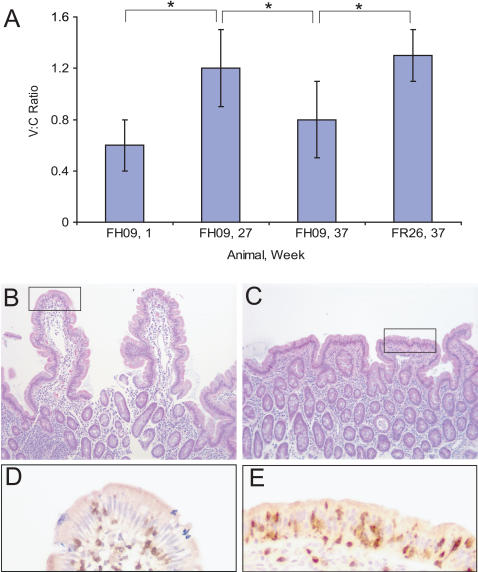
Gluten dependence of histological lesions in gluten-sensitive rhesus macaque FH09. (A) Morphometric analysis of villus height:crypt depth ratios from at least 4 different areas of distal duodenum in gluten-sensitive FH09 and control FR26 following dietary changes. Administration of a gluten-free diet increased the V:C ratio in FH09 at week 27 to a level that is statistically equivalent to that in FR26 (constant at all time points). Reintroduction of dietary gluten resulted in a drop in V:C ratio in FH09 (week 37) relative to that in FH09 on a gluten-free diet (week 27) and to that in FR26. *P<0.05. (B–C) H&E-stained duodenum at week 37 following 10 weeks of a gluten-containing diet. 100× magnification. (B) Control macaque FR26 exhibits normal villus architecture. (C) Gluten-sensitive macaque FH09 exhibits villus blunting. (D–E) Highlighted sections in B–C were examined by immunohistochemistry. 400× magnification. (D) Anti-CD3 staining in FR26 shows few CD3+ IELs (dark brown dots in epithelium). (E) Anti-CD3 staining in FH09 shows intraepithelial lymphocytosis.

Histological analysis of duodenal biopsies revealed a similarly beneficial effect of a gluten-free diet on mucosal architecture in FH09. The duodenal V:C ratios measured in FH09 at week 27, following 11 weeks on a gluten-free diet (diet #3), were significantly higher than those measured at week 1, on a gluten-containing diet (p<0.05), and were statistically equivalent to those measured in FR26 (Figure 3A). In contrast to FH09, the V:C ratios in FR26 did not change as a function of dietary changes (data not shown). The total number of CD3+ and CD103+ cells in the lamina propria and epithelium at week 27 was only ∼2-fold lower than it was at week 1 (data not shown). This suggests that many of the inflammatory cells that were present in the duodenum of FH09 on a gluten-containing diet persisted in the epithelium after clinical symptoms and plasma AGA levels were already normalized.

### Gluten-induced relapse

Reintroduction of dietary gluten was sufficient to reverse the effects of a gluten-free diet in FH09. At week 28, FH09 and FR26 were returned to the standard gluten-containing diet (Figure 2A). The clinical health of FH09 deteriorated within 2 weeks of the diet reversal, and by 6 weeks of gluten challenge FH09 was exhibiting symptoms of diarrhea, stomach bloating, and dehydration that were comparable in severity to those present prior to treatment with the gluten-free diet (Figure 2A). After 10 weeks of gluten challenge (week 37), dietary fat absorption in FH09 dropped to <70%, while that in FR26 macaque remained level at >80% (Table 3). Re-introduction of gluten-containing chow at week 28 was also associated with renewed and rapid seroconversion to gliadin: Both IgA and IgG AGA were by week 37 significantly elevated in FH09 above the AGA levels of FR26, despite identical dietary treatment (p<0.0001) (Figure 2B). At this point, FH09 displayed marked villus blunting and crypt hyperplasia, as well as increased intraepithelial lymphocyte (IEL) infiltration (Figure 3C and E), when compared to the rich villus architecture and relatively fewer IEL seen in the control, FR26 (Figure 3B and D). Remarkably, the difference between the V:C ratios from FH09 and FR26 at week 37 was significant at the p<0.05 level, as was the difference between the V:C ratios measured from FH09 in remission (week 27) and after relapse (week 37) (Figure 3A).

Although we were not able to continue collecting intestinal biopsies from additional macaques in this study, the gluten-dependency of clinical diarrhea and AGA observed in FH09 was corroborated in an additional experiments with gluten-sensitive and control animals [16].

The clinical, histological, and serological markers of disease in gluten-sensitive macaques are thus induced by ingestion of dietary gluten, and are abrogated by removal of gluten from the diet. Accordingly, we designate this condition simian gluten sensitivity, in analogy to that similar condition observed in human celiac disease patients. While it would be premature to proffer gluten-sensitive macaques as a bona fide animal model for celiac disease absent evidence of MHC (Mamu) class II association and of TG2 involvement, the gluten-inducible nature of signs and symptoms in these macaques provides an excellent *in vivo* system for studying oral glutenase efficacy as well as intestinal permeability toward gluten peptides under varied states of intestinal disrepair.

## Discussion

Chronic diarrhea is the primary cause of morbidity in colonies of captive non-human primates [18]. A number of infectious pathogens have been identified that can induce this condition [18], [26], [27], but the role of dietary antigens had not been previously investigated. We hypothesized that captive rhesus macaques exhibiting clinical diarrhea of non-infectious origin may be sensitive to gluten, a major source of protein in their formulated diet. A subpopulation of these animals presented with chronic diarrhea, stomach distention and blistering skin rashes, clinical symptoms that are also observed in classic human celiac disease, and in its dermatologic manifestation, dermatitis herpetiformis. In this study we characterized the clinical, histological, and serological characteristics of a small number of gluten-sensitive macaques. We hope to continue with similar, more extensive studies in the future.

Juvenile macaques appear to be especially prone to reacting adversely to dietary gluten. The majority of animals with chronic diarrhea of non-infectious origin had elevated levels of anti-gliadin IgG and IgA in their plasma. A number of these seropositive macaques exhibited marked villus blunting, crypt hyperplasia, and intraepithelial lymphocytosis in duodenal biopsies. Their clinical, histological and serological markers resolved upon administration of a gluten-free diet, and returned upon reintroduction of dietary gluten.

Previously proposed animal models for gluten sensitivity include non-human primates, gluten-fed rabbits, Irish setter dogs, and transgenic mice (Table 4). Prior documentation of celiac disease-like enteropathy in non-human primates is limited to two case reports, one in a single rhesus macaque necropsy [28], and another in a single cynomolgus monkey which improved on a gluten-free diet [29]. Elevated anti-gliadin IgG are observed in a majority of laboratory rabbits fed gluten as part of their standard diet [30] (MTB, unpublished results), but anti-gliadin IgA are not present (MTB, unpublished results) and these rabbits are apparently asymptomatic. Irish setter dogs with gluten-sensitive enteropathy are the best-studied natural animal model for gluten sensitivity, exhibiting both gluten-dependent diarrhea and histological lesions [31]–[33]. However, the absence of anti-gliadin and anti-TG2 antibodies as well as the lack of MHC class II linkage with disease in these animals precludes their use as a model for celiac disease [34], [35]. Finally, transgenic mouse models have been engineered to mimic celiac disease, most notably the NOD Ab° DQ8^+^ mouse, which expresses human DQ8 in an endogenous MHC class II-deficient (Ab°), autoimmune-prone (NOD) background [36]. These mice develop skin rashes with subcutaneous IgA deposits that are reminiscent of dermatitis herpetiformis, but have no gastrointestinal lesions or GI-related symptoms. Thus, rhesus macaques may represent a more complete model for studying celiac disease pathology than prior animal models.

**Table 4:** Comparison of celiac disease with proposed animal models for gluten sensitivity

					Serology				
		Clinical symptoms		Histology	anti-gliadin		anti-TG2/anti-endomysium		Genetics
Model system/disease	Propensity for gluten sensitivity	Diarrhea	Skin rash	Intestinal Lesions	IgG	IgA	IgG	IgA	MHC II association
Rabbit	natural	− A	− A	ND	+ A	− A	ND	ND	ND
Irish setter dog	natural	+ B	− B	+ B	− C	− C	ND^D^	ND^D^	− E
NOD Ab° DQ8^+^ mouse	transgenic	− F	+ F	− F	+ F	− F	−F	− F	+ (transgene) F
Juvenile rhesus macaque	natural	+	+	+	+	+	±^G^	−	ND^H^
Celiac disease	natural	+	+	+	+	+	+	+	+

A March 2003;

B Batt 1984, Batt 1987, Hall 1992;

C Hall 1992;

D Not tested for anti-TG2, but negative for anti-reticulin antibodies, unpublished data cited in Polvi 1998;

E Polvi 1997, Polvi 1998;

F Marietta 2004;

G Not observed in majority of clinically ill macaques, but anti-TG2 IgG antibodies were observed in gluten-sensitive macaque FH45 during gluten challenge with EP-B2 treatment (Bethune et al., in press);

H testing underway at TNPRC.

Two important issues must be addressed before the implications of this animal model can be fully appreciated. First, a Mamu class II association with simian gluten sensitivity must be identified, in analogy to the strong association of MHC class II haplotypes DQ2/DQ8 with human celiac disease. Testing for such a genetic association is currently underway. Preliminary results show that the two gluten-sensitive macaques studied extensively herein and in the accompanying report [16], FH09 and FH45, are of genotype DRB1*0303(12), DRB*1007 at the Mamu class II DRB(1) locus. Second, a humoral immune response directed against TG2 is enacted during active enteropathy in celiac disease patients, but is not observed in gluten-sensitive macaques. This may be because we used recombinant human TG2 as antigen in our ELISA to capture plasma antibodies raised against rhesus macaque TG2. Human and macaque TG2 share 95% protein sequence identity, but may present different epitope binding surfaces in three-dimensional space, attenuating antibody affinity in our assay. Another reason for this discrepancy might be that in macaques TG2-mediated deamidation of gluten epitopes is not required for their presentation by gluten-binding Mamu class II alleles. Finally, if the pathogenesis of macaque and human gluten-sensitivity are substantially shared, it may reflect the possibility that anti-TG2 antibodies do not have a significant pathogenic role in celiac disease. Regardless of the answers to the above unresolved issues, gluten-sensitive rhesus macaques are likely to be valuable for studying the pathogenesis and treatment of human gluten sensitivity.

## References

[1] Dicke WK, Weijers HA, Van De Kamer JH (1953). Coeliac disease. II. The presence in wheat of a factor having a deleterious effect in cases of coeliac disease.. Acta Paediatr.

[2] Alaedini A, Green PH (2005). Narrative review: celiac disease: understanding a complex autoimmune disorder.. Ann Intern Med.

[3] Green PH, Jabri B (2006). Celiac disease.. Annu Rev Med.

[4] Sollid LM, Markussen G, Ek J, Gjerde H, Vartdal F (1989). Evidence for a primary association of celiac disease to a particular HLA-DQ alpha/beta heterodimer.. J Exp Med.

[5] Spurkland A, Ingvarsson G, Falk ES, Knutsen I, Sollid LM (1997). Dermatitis herpetiformis and celiac disease are both primarily associated with the HLA-DQ (alpha 1*0501, beta 1*02) or the HLA-DQ (alpha 1*03, beta 1*0302) heterodimers.. Tissue Antigens.

[6] Sollid LM (2002). Coeliac disease: dissecting a complex inflammatory disorder.. Nat Rev Immunol.

[7] (1990). Revised criteria for diagnosis of coeliac disease. Report of Working Group of European Society of Paediatric Gastroenterology and Nutrition.. Arch Dis Child.

[8] Maki M (1995). The humoral immune system in coeliac disease.. Baillieres Clin Gastroenterol.

[9] Dieterich W, Ehnis T, Bauer M, Donner P, Volta U (1997). Identification of tissue transglutaminase as the autoantigen of celiac disease.. Nat Med.

[10] Johnston SD, Watson RG, McMillan SA, Sloan J, Love AH (1998). Coeliac disease detected by screening is not silent–simply unrecognized.. QJM.

[11] Rewers M (2005). Epidemiology of celiac disease: what are the prevalence, incidence, and progression of celiac disease?. Gastroenterology.

[12] Corrao G, Corazza GR, Bagnardi V, Brusco G, Ciacci C (2001). Mortality in patients with coeliac disease and their relatives: a cohort study.. Lancet.

[13] Sollid LM, Khosla C (2005). Future therapeutic options for celiac disease.. Nat Clin Pract Gastroenterol Hepatol.

[14] Stepniak D, Koning F (2006). Enzymatic gluten detoxification: the proof of the pudding is in the eating!. Trends Biotechnol.

[15] Cerf-Bensussan N, Matysiak-Budnik T, Cellier C, Heyman M (2007). Oral proteases: a new approach to managing coeliac disease.. Gut.

[16] Bethune MT, Khosla C, Sestak K (2008). Transepithelial transport and enzymatic detoxification of gluten in gluten-sensitive rhesus macaques.. PLoS ONE in press.

[17] Jandacek RJ, Heubi JE, Tso P (2004). A novel, noninvasive method for the measurement of intestinal fat absorption.. Gastroenterology.

[18] Sestak K, Merritt CK, Borda J, Saylor E, Schwamberger SR (2003). Infectious agent and immune response characteristics of chronic enterocolitis in captive rhesus macaques.. Infect Immun.

[19] Piper JL, Gray GM, Khosla C (2002). High selectivity of human tissue transglutaminase for immunoactive gliadin peptides: implications for celiac sprue.. Biochemistry.

[20] Borda JT, Alvarez X, Kondova I, Aye P, Simon MA (2004). Cell tropism of simian immunodeficiency virus in culture is not predictive of in vivo tropism or pathogenesis.. Am J Pathol.

[21] Ramesh G, Alvarez X, Borda JT, Aye PP, Lackner AA (2005). Visualizing cytokine-secreting cells in situ in the rhesus macaque model of chronic gut inflammation.. Clin Diagn Lab Immunol.

[22] Marsh MN (1992). Gluten, major histocompatibility complex, and the small intestine. A molecular and immunobiologic approach to the spectrum of gluten sensitivity (‘celiac sprue’).. Gastroenterology.

[23] Wahab PJ, Meijer JW, Mulder CJ (2002). Histologic follow-up of people with celiac disease on a gluten-free diet: slow and incomplete recovery.. Am J Clin Pathol.

[24] Schuppan D, Dennis MD, Kelly CP (2005). Celiac disease: epidemiology, pathogenesis, diagnosis, and nutritional management.. Nutr Clin Care.

[25] Shan L, Molberg O, Parrot I, Hausch F, Filiz F (2002). Structural basis for gluten intolerance in celiac sprue.. Science.

[26] Sestak K (2005). Chronic diarrhea and AIDS: insights into studies with non-human primates.. Curr HIV Res.

[27] Dassanayake RP, Zhou Y, Hinkley S, Stryker CJ, Plauche G (2005). Characterization of cytolethal distending toxin of campylobacter species isolated from captive macaque monkeys.. J Clin Microbiol.

[28] Harris RL, Streett JW, Morrow D, Lord PF (1984). Villus atrophy and malabsorption in a rhesus monkey.. Lab Anim Sci.

[29] Wagner JD, Jerome CP, Adams MR (1988). Gluten-sensitive enteropathy in a cynomolgus monkey.. Lab Anim Sci.

[30] March JB (2003). High antigliadin IgG titers in laboratory rabbits fed a wheat-containing diet: a model for celiac disease?. Dig Dis Sci.

[31] Batt RM, Carter MW, McLean L (1984). Morphological and biochemical studies of a naturally occurring enteropathy in the Irish setter dog: a comparison with coeliac disease in man.. Res Vet Sci.

[32] Batt RM, McLean L, Carter MW (1987). Sequential morphologic and biochemical studies of naturally occurring wheat-sensitive enteropathy in Irish setter dogs.. Dig Dis Sci.

[33] Hall EJ, Batt RM (1992). Dietary modulation of gluten sensitivity in a naturally occurring enteropathy of Irish setter dogs.. Gut.

[34] Polvi A, Garden OA, Elwood CM, Sorensen SH, Batt RM (1997). Canine major histocompatibility complex genes DQA and DQB in Irish setter dogs.. Tissue Antigens.

[35] Polvi A, Garden OA, Houlston RS, Maki M, Batt RM (1998). Genetic susceptibility to gluten sensitive enteropathy in Irish setter dogs is not linked to the major histocompatibility complex.. Tissue Antigens.

[36] Marietta E, Black K, Camilleri M, Krause P, Rogers RS (2004). A new model for dermatitis herpetiformis that uses HLA-DQ8 transgenic NOD mice.. J Clin Invest.

